# Enhancing epidural analgesia access during labor: a pilot study on the use of translated informational materials

**DOI:** 10.1186/s44158-025-00266-7

**Published:** 2025-07-24

**Authors:** Fabrizia Calabrese, Paolo Mele, Marta Stella, Alessandro De Cassai, Roberto Tozzi, Paolo Navalesi

**Affiliations:** 1 Institute of Anesthesia, University Hospital of Padua, Padua, Italy; 2 Department of Medicine, University of Padua, Padua, Italy; 3 Unit of Gynecology and Obstetrics, Department of Women and Children’s Health, University of Padua, Padua, Italy

Dear Editor,

International migration is a growing global phenomenon, resulting in an increasing number of immigrant patients seeking healthcare during labor, accounting for 20.1% of all parturients in Italy [1]. However, studies have shown that immigrant patients are less likely to receive epidural analgesia in labor (EAL) [2]. Several factors have been proposed to explain this lower access rate. These include medical staff paying less attention to immigrants' expressions of pain during delivery, language barriers that hinder effective communication about perinatal pain management options [3], and cultural differences in pain expression, which may lead to misinterpretation by healthcare providers [4].

To address the linguistic barriers faced by non-Italian-speaking patients at our institution, we introduced a new initiative in July 2024. At our institution, it is standard practice to obtain informed consent in the presence of a language mediator when patients face linguistic barriers. This approach helps prevent misunderstandings and ensures that patients fully understand the proposed procedures. In addition, we provide all patients with written informational materials outlining the indications, contraindications, procedural steps, potential risks and their management associated with EAL. This supports the patient in making a well-informed decision even after the consultation with the anesthesiologist. The translated informational material was designed not only to provide linguistically appropriate content but also to promote patient autonomy and reduce dependence on real-time interpreter availability. The handouts served as both a reinforcement of the anesthesiology consultation and a stand-alone educational tool that patients could review at their own pace, enabling more thoughtful, informed decision-making. However, it is important to note that until last year, this written material was available only in Italian. For this reason, we translated the written informational materials on EAL from Italian into Arabic, Chinese, English, French, Punjabi, and Russian. The translations of the written materials were performed by professional translators, followed by a back-translation process to ensure accuracy. The final versions were reviewed after a back-translation by anesthesiologists with expertise in EAL.

Starting on August 1, 2024, non-Italian-speaking patients undergoing pre-labor anesthesiology consultations were provided with these written translated materials. We hypothesized that offering additional information about EAL in patients’ native languages during anesthesiology consultations even if the attending anesthesiologist is not able to communicate with the patient in her mother-tongue could improve EAL access among this subgroup.

Following Institutional Review Board approval (529n/AO24 on 07/11/2024) and informed consent from the patients, we conducted a retrospective analysis of data from patients who gave birth between May 1, 2024, and October 31, 2024, considering that before August 1 2024 informational material was provided only in Italian and since that date informational written material was available also in other languages. Inclusion criteria encompassed patients who underwent labor, were evaluated by an anesthesiologist before hospital admission for delivery, and whose native language was not Italian.

Data are presented as median (first-third quartile) for continuous variables and as percentage (number) for categorical variables. Continuous variables were compared using the Wilcoxon test, while categorical variables were analyzed using the Chi-square test. A p-value of < 0.005 was considered statistically significant. Analysis was performed using R (version 4.4.2).

Over the study period we identified 60 eligible patients; however, 14 were excluded from the final analysis as 10 patients underwent programmed cesarean section, one patient was excluded due to illiteracy and could not receive the informative material and three declined to enter the analysis, leaving a final study population of 46 patients. Of these, 26 since the implementation of translated materials on August 1, 2024, while 20 were before this date (non-MT group). The groups were comparable in all characteristics except for previous pregnancy history (Table 1).

**Table 1:** Population characteristics

	Non Mother Tongue (*n* = 20)	Mother Tongue(*n* = 26)	*p*-value
Age, mean (SD)	29.5 (24.5—33.5)	29 (27.25—31.75)	0.911
BMI > 30, % (n)	40 (8)	46.2 (12)	0.676
Pre-pregnancy BMI > 30, % (n)	20 (4)	23.1 (6)	0.802
Pre-eclampsy, % (n)	0 (0)	3.8 (10)	0.375
Gestational diabetes, % (n)	15 (3)	23.1 (6)	0.493
Anesthesiology consultation GW, mean (SD)	36.5 (35—37)	37 (36.25—37.75)	0.149
Previous Pregnancy, % (n)	80 (16)	50 (13)	0.036*
Spoken Language, %(n)			0.319
Arabic	10 (2)	19 (5)	
Chinese	15 (3)	19 (5)	
English	15 (3)	23 (6)	
French	15 (3)	23 (6)	
Pungjabi	35 (7)	15 (4)	
Russian	10 (2)	0 (0)	

BMI Body Mass Index, GW Gestational week

^*^p-value < 0.05

Patients in the MT group had a higher rate of EA access (53.8%, *n* = 14) compared to the non-MT group (30%, *n* = 6), though this difference did not reach statistical significance (*p* = 0.105).

The lack of statistical significance is likely due to the limited sample size. Despite this, the observed trend suggests that providing translated informational materials may positively impact EA access among non-Italian-speaking patients and will serve as the foundation for a prospective controlled study aimed at further evaluating the effectiveness of this intervention. Moreover, these results, while preliminary, highlight the potential value of multilingual written materials as a low-cost, easily implementable tool to support equitable access to EAL. Unlike interpreter services, which are often limited in availability, especially during off-hours, translated handouts can be distributed systematically during prenatal visits or pre-admission consultations. As such, they represent a simple yet impactful strategy to enhance informed decision-making and reduce disparities in pain management during labor.

## Data Availability

Data analyzed in this study will be available upon reasonable request and IRB approval.
